# Road Traffic Injuries in South Africa: A Complex Global Health Crisis

**DOI:** 10.5334/aogh.4249

**Published:** 2024-04-05

**Authors:** Heike Geduld, Marion Sinclair, Elmin Steyn, Kathryn Chu

**Affiliations:** 1 Stellenbosch University, ZA

**Keywords:** Road Traffic Crashes, SDG 3.6, Road Safety, Safe Systems Approach

## Abstract

1.3 million people die each year as a result of Road traffic crashes. Road Traffic Injuries are a global health crisis with 90% of global deaths affecting LMICs. Sustainable Development Goal 3.6 focuses on reducing road injury and death. The global plan is based on the Safe Systems approach.

In South Africa, the burden of crashes on the health system and society is particularly high with a population death rate of 20.7 per 100 000 population. Understanding local context and culture is critical. Rurality, distorted urban planning, higher travel exposure and alcohol usage disproportionately impact racial and ethnic minorities. Pedestrian safety is a key priority.

There is a critical need for the global health community to take an active role in advocacy in order to achieve SDG 3.6 by 2030.

It is a sobering fact that over 1.3 million people die each year as a result of road traffic crashes – on average one fatality every 23 seconds – with millions more injured or disabled. Road traffic crashes are the leading global cause of death in children and young adults aged 5–29 years [1]. Road traffic injuries are a global crisis and more specifically, a global health crisis. While all parts of the world experience traffic fatalities, LMICs are disproportionately represented, with more than 90% of global deaths occurring in these regions. Road crashes significantly affect those in the lowest socio-economics strata: the poor, rural populations, and racial and ethnic minorities [2]. Frustratingly, many traffic deaths are preventable and with more concerted effort there is significant room to reduce these figures, particularly in regions where road safety interventions have been poor or absent.

The WHO is the lead agency for the global response to road traffic crashes and yet Health as a sector has not yet played a significant role in addressing the causes of and advocating for solutions to road traffic crashes. Sustainable Development Goal 3.6 – to reduce road injuries and death – was the driver for the Decade of Action for Road Safety 2010–2020 which was reset for 2020–2030. The UN General Assembly Resolution 74/299 declared a target to reduce global road traffic injuries and deaths by at least 50% [3]. The global plan is based on the Safe System Approach, conceptualising that humans are both fragile (vulnerable to kinetic forces and should be protected) and fallible (error prone) and so roads should be self-explanatory and forgiving [3]. Current road safety practice is based largely on managing and identifying the interactions between multiple components of the transport system including road infrastructure, speed management, vehicle standards (Road and Traffic Factors); and road user behaviour (Human Factors). While the safe system approach encourages interdisciplinary collaboration, in reality most of the action around road safety still resides in the field of transportation engineering. This is in spite of the fact that the burden of the consequences of poor road safety rests largely on the health community [4].

In South Africa the burden of crashes on the health system and society is particularly high. In 2022, 12,436 people died on the road; a population death rate of 20.7 per 100,000 population. In addition, over 100,000 people are seriously injured per year. Pedestrians account for 43% of those killed on the road. Males account for 75% of fatalities [5].

In spite of being a signatory to the UN Decade of Action there is still a lack of visible cross-disciplinary leadership in South Africa around reducing the high numbers of road related casualties. Even with annual public awareness programs during holiday seasons, the numbers of crashes remain high. This sense that the SA crash levels are somehow “normal” undermines the potential for individual or group interventions that could make a real impact – these include speed limit reduction and zero alcohol limits. The lack of awareness of the factors that lead to high crash risks among the general populace suggest that there is an important role to play for healthcare providers in road safety advocacy [4].

One of the critical aspects of road safety strategies is to understand the local context and culture to prioritise and design appropriate interventions. Sukhai et al., in describing the fatality risk and issues of inequity among vulnerable road users in South Africa, demonstrated a disproportional impact of road injuries on racial and ethnic minorities [2]. Vulnerability is conferred by road use, traffic and social environments including rurality, socio-economic deprivation, travel exposure, and crime and violence. The distorted urban planning legacy of Apartheid has created areas of socio-spatial disparity which has directly increased the vulnerability for certain populations to road crashes. This “area-level deprivation” presents itself in terms of higher travel exposure, inadequate safe provisioning for pedestrians and informal commercial activities on the roadside pushing pedestrians and traffic into the same spaces. Pedestrian safety should be the focus in South Africa and other LMICs with similar road use, as well as socio-economic and traffic patterns.

The impact of alcohol on road safety in South Africa is also significant. South Africa ranks sixth globally in the amount of alcohol consumed per drinker, and 6.4% of all deaths are associated with alcohol, and once again this disproportionately impacts those from lower socio-economic groups. Although the accuracy of crash data is disputed, it is estimated at least 27% of driver-error attributed fatal crashes are caused by alcohol intoxication [6]. Early legislative steps have been made recently in the move towards a zero tolerance system (zero blood alcohol limit); however, this may be limited by the lack of political will, sociocultural forces, a dominant alcohol industry and inconsistent enforcement and prosecution.

Road traffic crashes are a global health crisis that has been ignored for too long. There are multiple interventions to road infrastructure, vehicle safety and road user behaviour that can make the road system safer. There is a critical need for the global health community in South Africa and elsewhere to take an active role in advocacy if there is to be any hope of achieving SDG 3.6, and with it a 50% reduction in road deaths and injuries by 2030.
